# Contrasting Responses of Rhizosphere Bacterial and Fungal Communities of *Paeonia ludlowii* to Habitat Transition

**DOI:** 10.3390/microorganisms14091919

**Published:** 2026-08-31

**Authors:** Ruiwen Zhang, Xiazhen Yao, Zhen Xing

**Affiliations:** College of Forestry and Grassland, Xizang Agricultural and Animal Husbandry University, Nyingchi 860000, China; zrw9790@163.com (R.Z.); xztibetan@xza.edu.cn (Z.X.)

**Keywords:** *Paeonia ludlowii*, rhizosphere microbiome, bacterial and fungal communities, habitat transition, community reorganization

## Abstract

Habitat transition can reorganize plant-associated microbial communities, yet whether rhizosphere bacteria and fungi respond similarly to the transition from native to introduced habitats remains unclear. Here, we investigated this question in the endangered plant *Paeonia ludlowii* by comparing rhizosphere bacterial and fungal communities across three native and two introduced sites in Xizang. Both bacterial and fungal communities showed significant site-associated differentiation and strongly concordant spatial patterns. However, their responses differed markedly: bacterial richness remained relatively stable despite compositional turnover, whereas fungal communities showed reduced richness at the Lhasa introduction site, lower OTU sharing among habitats, and significantly greater divergence from native assemblages than bacterial communities. Soil pH and available phosphorus were the environmental variables most consistently associated with differentiation in both microbial groups. These findings demonstrate that rhizosphere bacteria and fungi exhibit concordant spatial differentiation but contrasting responses to habitat transition, with fungal communities showing greater departure from native states. This contrast highlights the importance of considering bacterial and fungal communities jointly when evaluating rhizosphere reorganization during the introduction and ex situ conservation of endangered plants.

## 1. Introduction

Global change and human activities are continuously altering the environments in which plants survive [1]. Habitat fragmentation, climate change, and anthropogenic disturbances have led to declines in numerous plant populations, posing new challenges for the conservation of endangered plants [2]. In recent years, studies of the plant microbiome have shown that plants do not exist in isolation but instead form complex plant–microbe symbiotic systems with their associated microorganisms [3]. The rhizosphere is a major interface for interactions between plants and the soil environment [4], and its microbial communities contribute to nutrient cycling, promote plant growth, and enhance plant tolerance to environmental stress [5]. From a microbial ecological perspective, soil and plant-associated microbiota are increasingly recognized as important components of ecosystem restoration and biodiversity conservation [6]. Research on plant-associated microorganisms has gradually shifted from early descriptions of community composition toward understanding microbial community assembly mechanisms and ecological functions [7,8]. Accordingly, integrating microbiome ecology into plant conservation and ecological restoration has become an increasingly important research direction [9].

Over the course of long-term evolution, plants typically establish stable associations with microbial communities in their surrounding environments [10]. Rhizosphere microbial communities are jointly shaped by multiple factors, including host selection [11], soil properties [12], and environmental conditions [13], and exhibit varying degrees of host specificity and environmental adaptation [14]. However, when plants undergo ex situ conservation, introduction, or habitat transition, changes in environmental conditions may alter the available microbial pool, leading to the reassembly of rhizosphere microbial communities. Changes in environmental filtering can substantially influence microbial community assembly and may cause microbial communities to diverge from their original composition [15]. Previous studies have shown that environmental change can reshape plant-associated microbial communities and alter microbiome functions related to plant resource acquisition and stress responses [16]. Bacterial and fungal communities may respond differently to environmental change because they are shaped by distinct combinations of host selection, soil properties, and ecological interactions [17,18]. Therefore, simultaneously examining changes in rhizosphere bacterial and fungal communities is important for understanding microbial responses associated with plant habitat transitions.

*Paeonia ludlowii* is a rare and endangered woody peony endemic to China and is mainly distributed in the Yarlung Zangbo River basin on the Qinghai–Tibet Plateau. Recent studies have begun to characterize the microbial communities associated with *P. ludlowii*, including its endophytic and rhizosphere bacterial and fungal communities. Lu et al. [19] characterized the endophytic and rhizosphere bacterial communities associated with *P. ludlowii*, while Gao et al. [20] characterized rhizosphere bacterial, fungal, and arbuscular mycorrhizal fungal communities of introduced *P. ludlowii*. Qiao et al. [21] further demonstrated clear differences in rhizosphere soil fungal communities between wild and cultivated *P. ludlowii*. However, systematic understanding remains limited regarding how rhizosphere soil bacterial and fungal communities respond jointly during the transition of *P. ludlowii* from its native habitat to new environments, whether these microbial groups exhibit distinct ecological response patterns, and to what extent microbial communities in introduced habitats diverge from those in native habitats. Therefore, from the perspective of habitat transition, this study compares changes in rhizosphere soil bacterial and fungal communities across native and introduced habitats and evaluates their differential patterns of community reassembly and divergence from native communities.

## 2. Materials and Methods

### 2.1. Study Sites and Soil Sampling

Rhizosphere soil was collected from wild native populations and introduction sites in Tibet (Table 1). Native populations were located in Qunigongga Village, Mirui Township, Bayi District, Nyingchi; Caimen Village, Zaxiraodeng Township, Milin; and Qinmaduo, Milin Town, Milin. Sampling was conducted on 11, 12, and 13 May 2026, respectively. Introduction sites were the botanical garden of the Plateau Ecology Institute, Tibet Agricultural and Animal Husbandry University, Bayi District, Nyingchi ($29^{\circ }39^{\prime }\;N,\;94^{\circ }21^{\prime }\;E$), and the Agricultural Science Research Station of the Tibet Academy of Agricultural and Animal Husbandry Sciences, Lhasa ($29.64^{\circ }\;N,\;91.11^{\circ }\;E$), sampled on 16 and 20 May 2026, respectively. Because *P. ludlowii* is a rare and endangered species with few wild populations, only two established introduction bases in Tibet (Nyingchi and Lhasa) were available for long-term observation and were therefore included as representative sites. Precise coordinates of the wild populations are not disclosed because of conservation concerns.

At each site, five flowering plants with similar vigor and no visible pests or disease were selected at least 3m apart. Surface litter and debris were removed, and soil adhering to roots was collected from the 0–20cm layer. Loosely attached soil was gently shaken off, and the soil remaining tightly attached to the root surface was collected as rhizosphere soil. Subsamples taken around the same plant were pooled to form one biological sample. Three composite surface-soil samples were additionally collected from the native region, the Nyingchi introduction site, and the Lhasa introduction site as background references. Because these background samples lacked biological replication, they were used descriptively only and were not included in significance tests. Sampling holes were backfilled immediately to minimize disturbance to plants and native habitats.

### 2.2. Soil Physicochemical and Molecular Analyses

Gravimetric water content was determined on fresh soil by oven drying at 105 °C. The remaining soil was air-dried, passed through a 2mm sieve, and analyzed for pH, soil organic matter (SOM), total nitrogen (TN), available phosphorus (AP), and available potassium (AK). Soil pH was measured using a Sartorius PB-10 benchtop pH meter (Sartorius, Göttingen, Germany) SOM was determined by potassium dichromate oxidation, TN by the Kjeldahl method, AP by extraction followed by colorimetry, and AK by ammonium acetate extraction followed by flame photometry.

Frozen soil samples were thawed on ice and thoroughly mixed. Approximately 200 mg of rhizosphere soil was transferred to a grinding tube containing 750 μL of Lysis Buffer A and 90 μL of Lysis Buffer B and mechanically homogenized using a grinder according to the manufacturer’s instructions. Total genomic DNA was subsequently extracted using the YH-soil DNA Extraction Kit (Shanghai Yuhua, Shanghai, China) and stored at −20 °C. DNA concentration and purity were assessed using a NanoDrop 2000 spectrophotometer (Thermo Fisher Scientific, Waltham, MA, USA), with $A_{260}/A_{280}$ ratios of 1.8–2.0 and $A_{260}/A_{230}$ ratios ≥1.8 considered acceptable. DNA integrity was evaluated by electrophoresis of 3μL DNA on a 1% agarose gel at $5\;V\;{\mathrm{cm}}^{-1}$ for 20 min, and only samples showing clear, nondegraded bands were used.

The bacterial 16S rRNA V4 region was amplified using primers 515F (5′-GTGCCAGCMGCCGCGGTAA-3′) and 806R (5′-GGACTACNVGGGTWTCTAAT-3′).

The fungal ITS2 region was amplified using primers gITS7 (5′-GTGARTCATCGARTCTTTTG-3′) and ITS4 (5′-TCCTCCGCTTTATTGATATGC-3′).

PCR amplification was performed in a total reaction volume of 20μL. The bacterial and fungal amplification reactions used the same thermal profile, except for the number of amplification cycles. Both reactions included an initial denaturation at 95 °C for 3 min, followed by denaturation at 95 °C for 30 s, annealing at 55 °C for 30 s, and extension at 72 °C for 45 s, with a final extension at 72 °C for 10 min and holding at 10 °C. The bacterial 16S rRNA V4 region was amplified for 27 cycles, whereas the fungal ITS2 region was amplified for 33 cycles.

Amplicons were evaluated by electrophoresis of 3μL product on 2% agarose gels at $5\;V\;{\mathrm{cm}}^{-1}$ for 20 min. Products of the expected size without nonspecific bands were purified, quantified, and used for library construction. Sequencing was performed by Majorbio Bio-Pharm Technology Co., Ltd. (Shanghai, China) on an Illumina NextSeq 2000 platform (Illumina, San Diego, CA, USA). Raw sequence data have been deposited in the NCBI Sequence Read Archive under BioProject accession PRJNA1498593.

### 2.3. Bioinformatics and Statistical Analyses

Raw reads were quality filtered using fastp (v0.19.6) [22], paired-end reads were merged using FLASH (v1.2.7) [23], and operational taxonomic units (OTUs) were clustered at 97% sequence similarity using UPARSE (USEARCH v11), with chimeric sequences removed [24]. Taxonomic assignments were generated using the RDP Classifier (v2.2) [25] against SILVA release 138.2 for bacteria and UNITE version 9.0 for fungi. To minimize bias associated with sequencing depth, bacterial and fungal OTU tables were rarefied to 48,387 and 49,695 sequences per sample, respectively.

Alpha diversity was characterized using observed OTU richness, Chao1, Good’s coverage, Shannon, inverse Simpson, and Pielou’s evenness indices. Principal-coordinate analysis (PCoA), hierarchical clustering, and permutational multivariate analysis of variance (PERMANOVA) were based on Bray–Curtis dissimilarities; PERMANOVA used 999 permutations. Concordance between bacterial and fungal communities was evaluated using Mantel and Procrustes analyses, with 9999 permutations for PROTEST. The 15 samples from MR, CM, and ML were used as the native reference set. For each LZ and LS sample, divergence from native communities was quantified as its mean Bray–Curtis dissimilarity to all native samples. Independent-samples Wilcoxon rank-sum tests were used to compare LZ and LS, whereas exact paired Wilcoxon signed-rank tests compared bacterial and fungal divergence within the same sample. Differentially abundant taxa were identified using LEfSe with an LDA score cutoff of 4.0. Core genera were defined as taxa with a prevalence ≥80% and a mean relative abundance ≥0.1%. Associations among soil properties, community structure, and core genera were assessed using db-RDA, Mantel tests, and Spearman correlations. The three composite surface-soil samples were used only to describe background soil conditions and were excluded from inferential and microbial community analyses.

Data were processed in Microsoft Excel 2016, IBM SPSS Statistics 27, and R version 4.4.1. One-way analysis of variance followed by Tukey’s honestly significant difference test was used for among-site comparisons, with p<0.05 considered statistically significant.

## 3. Results

### 3.1. Soil Physicochemical Properties Among Sites

Rhizosphere soil pH, SOM, TN, and AP differed significantly among sites (p<0.05), whereas AK (p=0.069) and gravimetric water content (p=0.435) did not. Soil conditions ranged from slightly acidic to slightly alkaline (Table 2). pH was highest at LS and lowest at LZ, while MR, CM, and ML were near neutral. Thus, pH varied more strongly between the two introduction sites than among the three native sites. SOM and TN showed similar patterns, with the highest values at CM and the lowest at LS. AP was highest at ML, followed by LS. Mean water content was highest at LS, although the among-site difference was not significant. Overall, LS was characterized by alkaline soil and relatively low SOM, TN, and AK, whereas ML showed high AP, indicating that differences among native and introduction sites reflected contrasting nutrient profiles rather than a single fertility gradient. Among the native sites, CM had the highest SOM and TN, ML had the highest AP, and MR had the highest mean AK. At LZ, soil pH was lowest and SOM and TN were intermediate. At LS, pH and AP were relatively high, whereas SOM, TN, and AK were relatively low, indicating an imbalanced nutrient profile.

The three composite surface-soil samples also indicated differences in background conditions. The composite native-soil sample had a pH of 7.28 and contained $48.46\;g\;{\mathrm{kg}}^{-1}$ SOM, $2.30\;g\;{\mathrm{kg}}^{-1}$ TN, $74.75\;\mathrm{mg}\;{\mathrm{kg}}^{-1}$ AP, and $354.58\;\mathrm{mg}\;{\mathrm{kg}}^{-1}$ AK. The composite soil from the Nyingchi introduction site was acidic (pH 5.70) and had relatively low AP and AK, whereas the composite soil from the Lhasa introduction site was alkaline (pH 7.83), with high AP but relatively low SOM and TN.

### 3.2. OTU Composition and α Diversity

**Figure 1:**
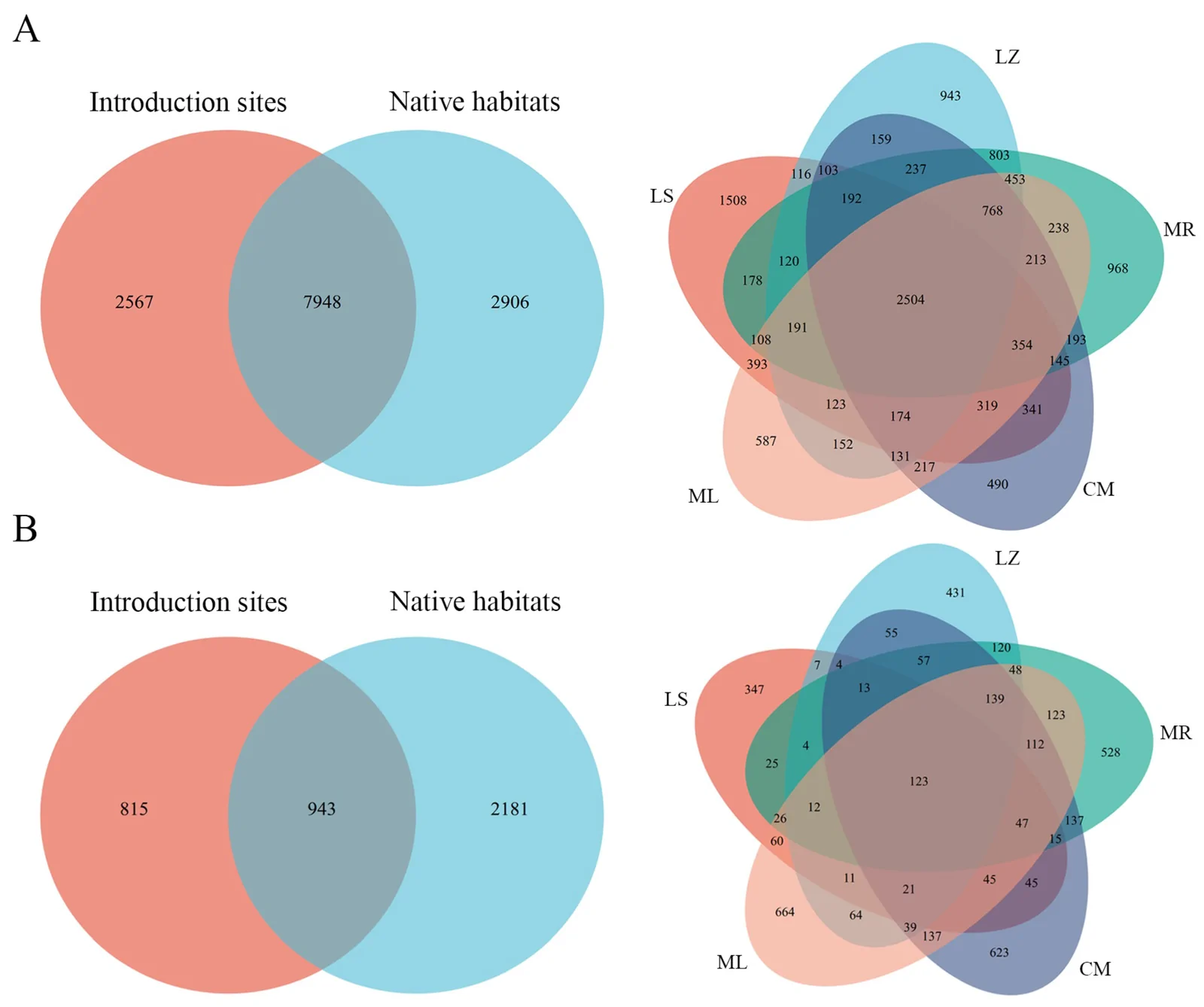
Venn diagrams of unique and shared bacterial (**A**) and fungal (**B**) OTUs in rhizosphere soil from native and introduction sites.

Sequencing yielded 1,935,331 optimized bacterial sequences, of which 1,834,601 passed quality filtering, with a mean length of approximately 255 bp. ITS sequencing yielded 1,821,577 optimized sequences, of which 1,799,090 passed quality filtering, with a mean length of approximately 282 bp. Rarefaction curves approached asymptotes for both bacteria and fungi, indicating adequate sampling of the dominant microbial taxa. When samples were pooled into native and introduction groups, 13,421 bacterial OTUs were detected, of which 7948 (59.22%) were shared (Figure 1). Across all five sites, 2504 bacterial OTUs were shared by MR, CM, ML, LZ, and LS, indicating a substantial shared bacterial component alongside site-specific taxa. Fungal OTUs showed a lower shared proportion. Across the native and introduction groups, 3939 fungal OTUs were detected; 943 (23.94%) were shared, 815 (20.69%) were unique to introduction sites, and 2181 (55.37%) were unique to native sites. Only 123 fungal OTUs were shared across all five sites. Relative to bacteria, fungi therefore exhibited a smaller shared OTU pool and greater site specificity, suggesting stronger sensitivity to local habitat and spatial heterogeneity.

Bacterial α-diversity varied little among sites (Table 3). Observed OTU richness, Chao1, Good’s coverage, and Shannon diversity did not differ significantly among the five sites (p>0.05), indicating relatively stable bacterial richness and overall diversity. Inverse Simpson and Pielou’s evenness indices differed significantly among sites (p<0.05), indicating localized changes in dominance and evenness. Fungal α-diversity showed a stronger site-related pattern. Observed OTU richness and Chao1 differed significantly among sites (p<0.001), and both were lower at LS than at MR, CM, ML, and LZ. Good’s coverage, Shannon diversity, inverse Simpson, and Pielou’s evenness did not differ significantly (p>0.05). Thus, bacterial richness remained relatively stable, whereas fungal richness was reduced at LS.

### 3.3. Rhizosphere Bacterial and Fungal Community Composition Across Sampling Sites

At the genus level, rhizosphere bacterial communities across the five sampling sites (MR, CM, ML, LZ, and LS) were characterized by a broadly similar set of dominant taxa, although their relative abundances varied among sites. The major bacterial genera included *Niallia*, *Bradyrhizobium*, *Arthrobacter*, *Pseudomonas*, and *Sphingomonas*. Among these, *Niallia* showed relatively higher abundances at ML and MR, *Bradyrhizobium* was more abundant at LZ, *Arthrobacter* was relatively more abundant at MR and CM, whereas *Pseudomonas* was particularly prominent at LS. Overall, the dominant bacterial genera were broadly shared across the five sampling sites, and differences in community composition were mainly characterized by shifts in the relative abundances of these shared dominant genera rather than by pronounced replacement of dominant taxa.

In contrast, fungal communities exhibited more pronounced site-associated variation in genus-level composition. The major fungal genera across the five sampling sites included *Solicoccozyma*, *Tausonia*, *Penicillium*, *Exophiala*, *Mortierella*, *Cladosporium*, and *Fusarium*, with their relative contributions varying among sites. In particular, *Tausonia* was prominent at LS, *Exophiala* showed a relatively high abundance at LZ, and *Penicillium* was more abundant at MR, whereas *Solicoccozyma* showed relatively higher abundances at CM and ML. Overall, compared with bacterial communities, the relative abundance profiles of dominant fungal genera varied more markedly among MR, CM, ML, LZ, and LS, indicating stronger site-associated compositional differentiation in fungal communities.

### 3.4. Overall Community Differentiation

**Figure 2:**
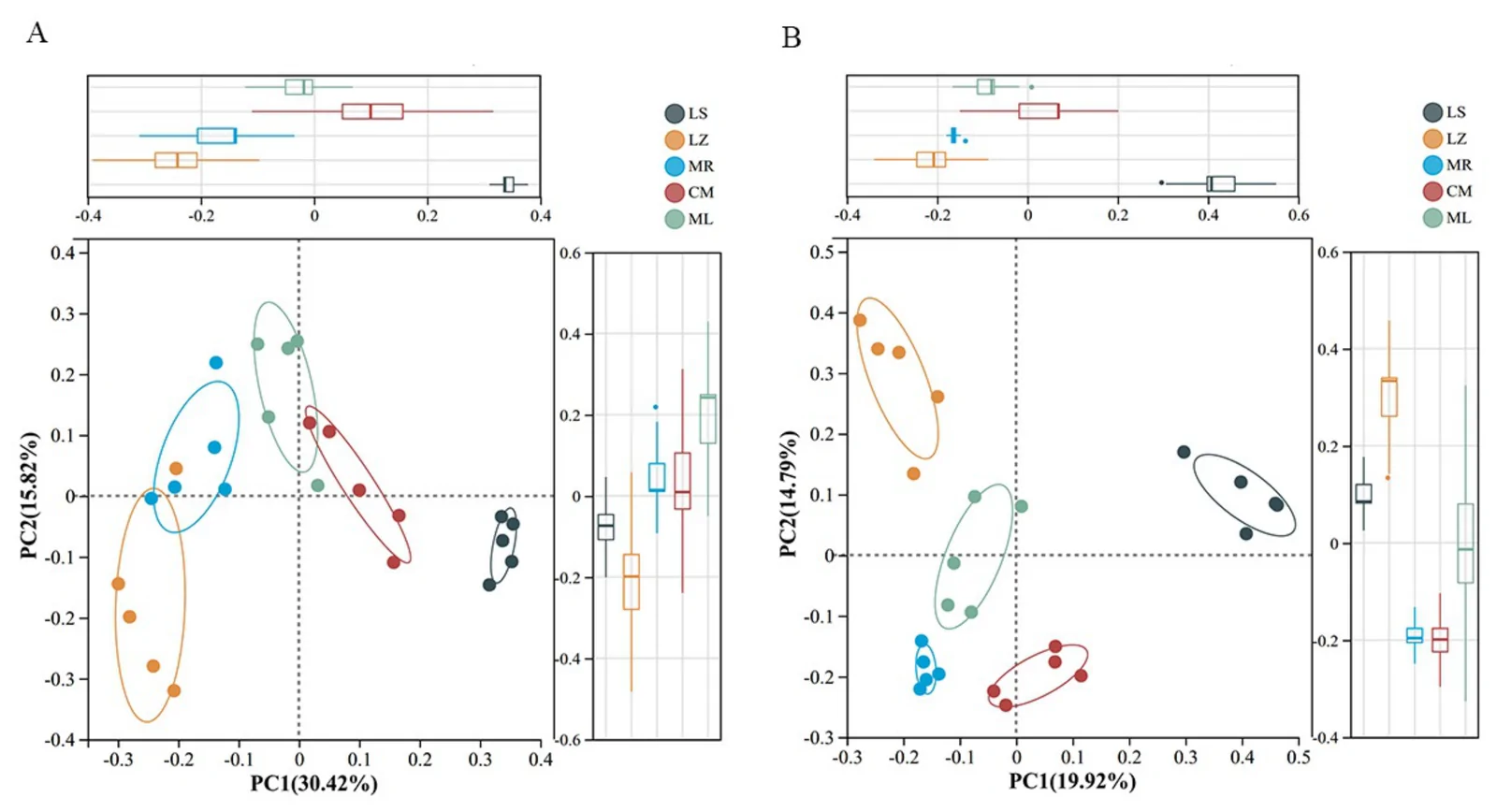
Principal-coordinate analysis of rhizosphere bacterial (**A**) and fungal (**B**) communities based on Bray–Curtis dissimilarities.

PCoA based on Bray–Curtis dissimilarities showed clear site-associated differentiation in both bacterial and fungal communities (Figure 2). For bacteria, PCoA1 and PCoA2 explained 30.42% and 15.82% of the variation, respectively, and community composition differed significantly among the five sites (PERMANOVA, $R^{2}=0.52278$, p=0.001). LS samples were positioned primarily on the positive side of PCoA1 and were clearly separated from the other four sites. Fungal communities also differed among sites: PCoA1 and PCoA2 explained 19.92% and 14.79% of the variation, respectively, and PERMANOVA indicated significant differences among the five sites ($R^{2}=0.45564$, p=0.001). LS fungal communities were clearly separated from those at the other sites. Direct comparison of the two introduction sites also showed significant differentiation. For bacteria, PCoA1 and PCoA2 explained 57.34% and 14.59% of the variation, respectively, and LZ and LS differed significantly ($R^{2}=0.56575$, p=0.015). For fungi, PCoA1 and PCoA2 explained 42.53% and 15.33% of the variation, respectively, and LZ and LS were also significantly separated ($R^{2}=0.41313$, p=0.015).

**Figure 3:**
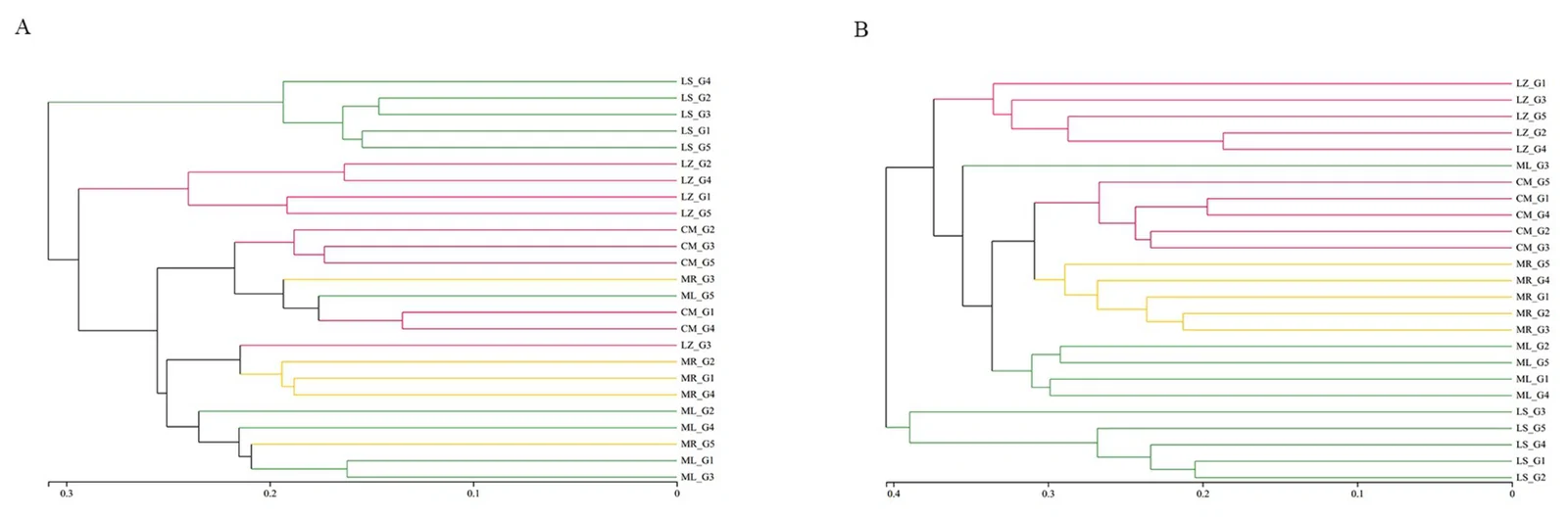
Hierarchical clustering of rhizosphere bacterial (**A**) and fungal (**B**) communities across sampling sites. Different branch colors indicate distinct clusters identified by hierarchical clustering and do not represent predefined sampling-site groups.

Hierarchical clustering supported the site-associated patterns observed in the PCoA (Figure 3). Bacterial communities from LS formed a relatively distinct cluster, whereas fungal communities from LZ and LS formed separate branches, indicating that the two introduction sites harbored distinct rhizosphere fungal assemblages.

### 3.5. Concordance Between Bacterial and Fungal Communities and Divergence from Native Communities

**Figure 4:**
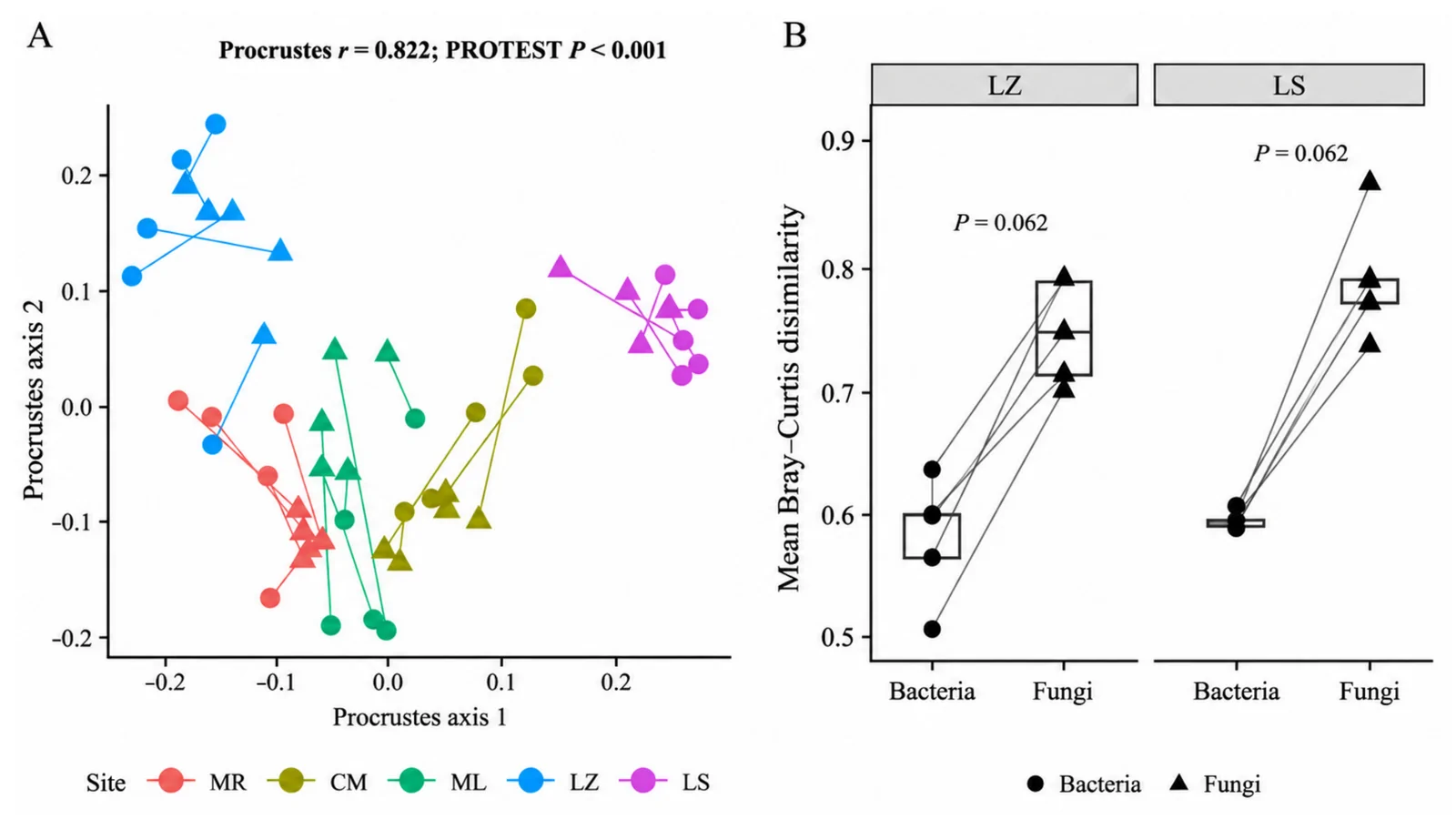
Concordance between bacterial and fungal community ordinations and their divergence from native communities in the rhizosphere of *Paeonia ludlowii*. (**A**) Procrustes analysis of bacterial and fungal community ordinations. (**B**) Mean Bray–Curtis dissimilarity of bacterial and fungal communities at LZ and LS relative to native samples. Lines connect paired bacterial and fungal observations from the same sample; boxes represent the interquartile range, and horizontal lines indicate medians.

Bray–Curtis dissimilarity matrices for bacterial and fungal communities were positively correlated (Mantel r=0.747, p<0.001). Procrustes analysis further showed significant concordance between the two ordination configurations (r=0.822, PROTEST p<0.001; Figure 4), indicating that bacterial and fungal communities exhibited broadly similar spatial differentiation among sites. Using the 15 samples from the three native sites (MR, CM, and ML) as a reference, we calculated the mean Bray–Curtis dissimilarity of each LZ and LS sample to all native samples as a measure of divergence from native communities. At LZ, mean divergence was 0.573±0.050 for bacteria and 0.748±0.044 for fungi; at LS, the corresponding values were 0.587±0.008 and 0.791±0.051. Divergence did not differ between LZ and LS for either bacteria (p=1.000) or fungi (p=0.421). Within each introduction site, fungal divergence was consistently greater than bacterial divergence, although site-specific exact paired Wilcoxon tests were not significant (both p=0.062). When all 10 introduction-site samples were analyzed together, fungal divergence was significantly greater than bacterial divergence (paired Wilcoxon test, p=0.002). Thus, bacterial and fungal communities showed concordant spatial differentiation, but fungal communities exhibited greater divergence from native communities.

### 3.6. Differentially Abundant Fungal Taxa Among Sites

**Figure 5:**
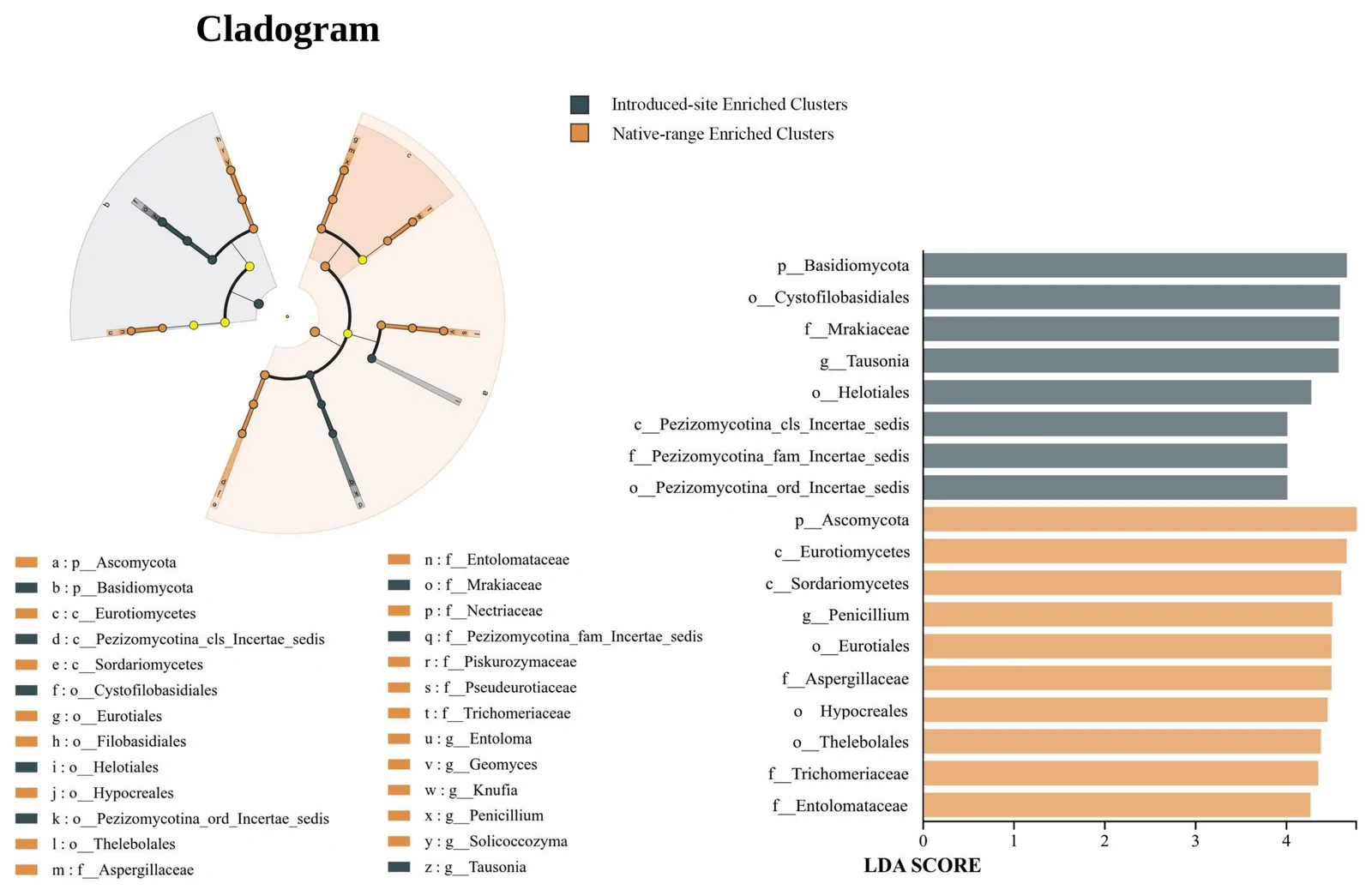
LEfSe analysis of rhizosphere fungal communities at native and introduction sites (LDA score ≥4.0). Yellow nodes in the cladogram indicate taxa without significant differences between groups, whereas colored nodes indicate taxa significantly enriched in the corresponding groups.

**Figure 6:**
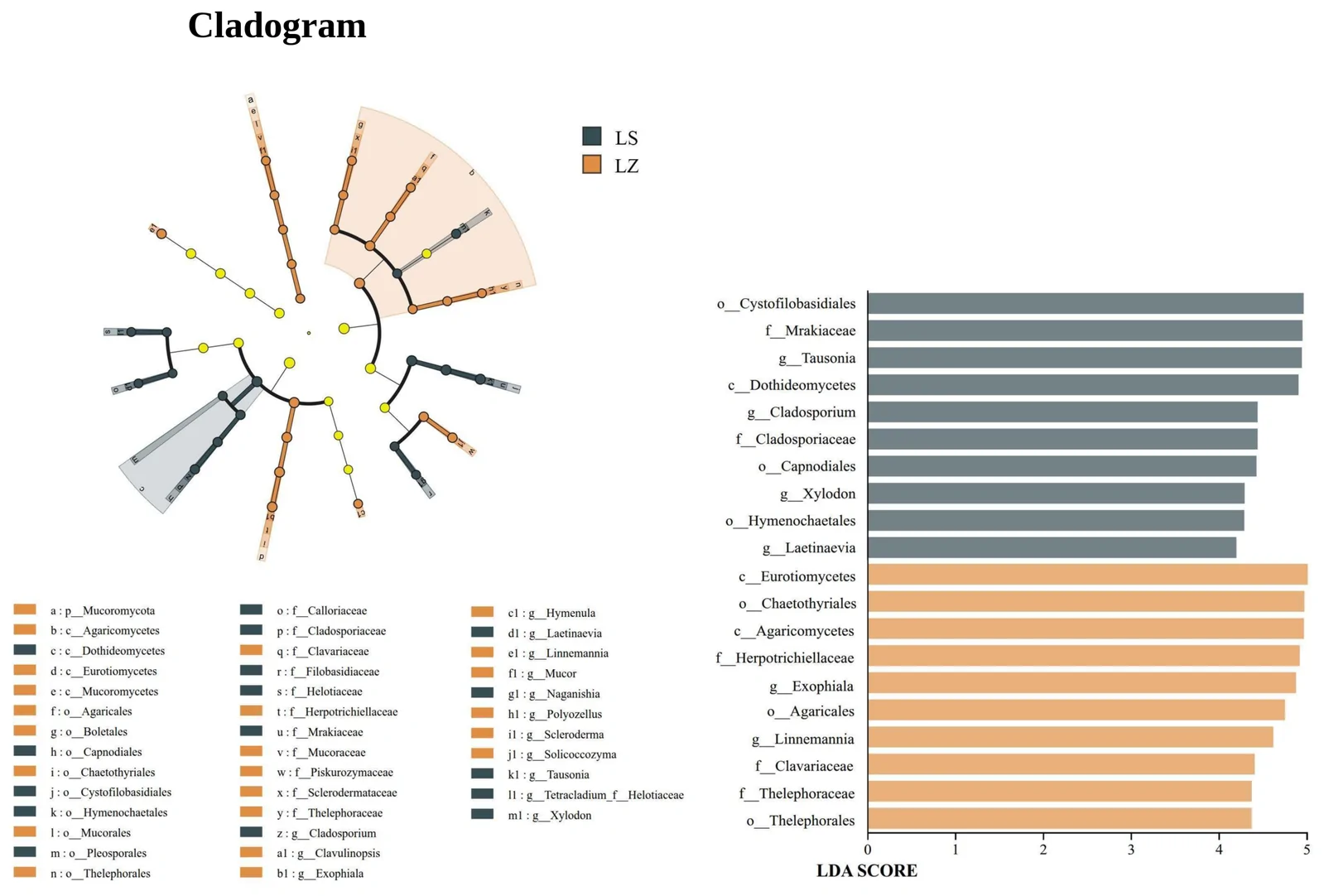
LEfSe analysis of rhizosphere fungal communities at the two introduction sites (LDA score ≥4.0). Yellow nodes in the cladogram indicate taxa without significant differences between groups, whereas colored nodes indicate taxa significantly enriched in the corresponding groups.

LEfSe identified distinct fungal indicator taxa between native and introduction sites (Figure 5). Basidiomycota, Cystofilobasidiales, Mrakiaceae, and *Tausonia* were enriched at the introduction sites, whereas Ascomycota, Eurotiomycetes, Sordariomycetes, *Penicillium*, Eurotiales, and Aspergillaceae were enriched at the native sites. Comparison of the two introduction sites (Figure 6) showed that Cystofilobasidiales, *Tausonia*, Dothideomycetes, and *Cladosporium* were enriched at LS, whereas Eurotiomycetes, Chaetothyriales, *Exophiala*, *Linnemannia*, and several agaric taxa were enriched at LZ. These results indicate that native and introduction sites, as well as the two introduction sites, were characterized by distinct fungal indicator assemblages.

### 3.7. Associations of Soil Properties and Elevation with Microbial Community Structure

**Figure 7:**
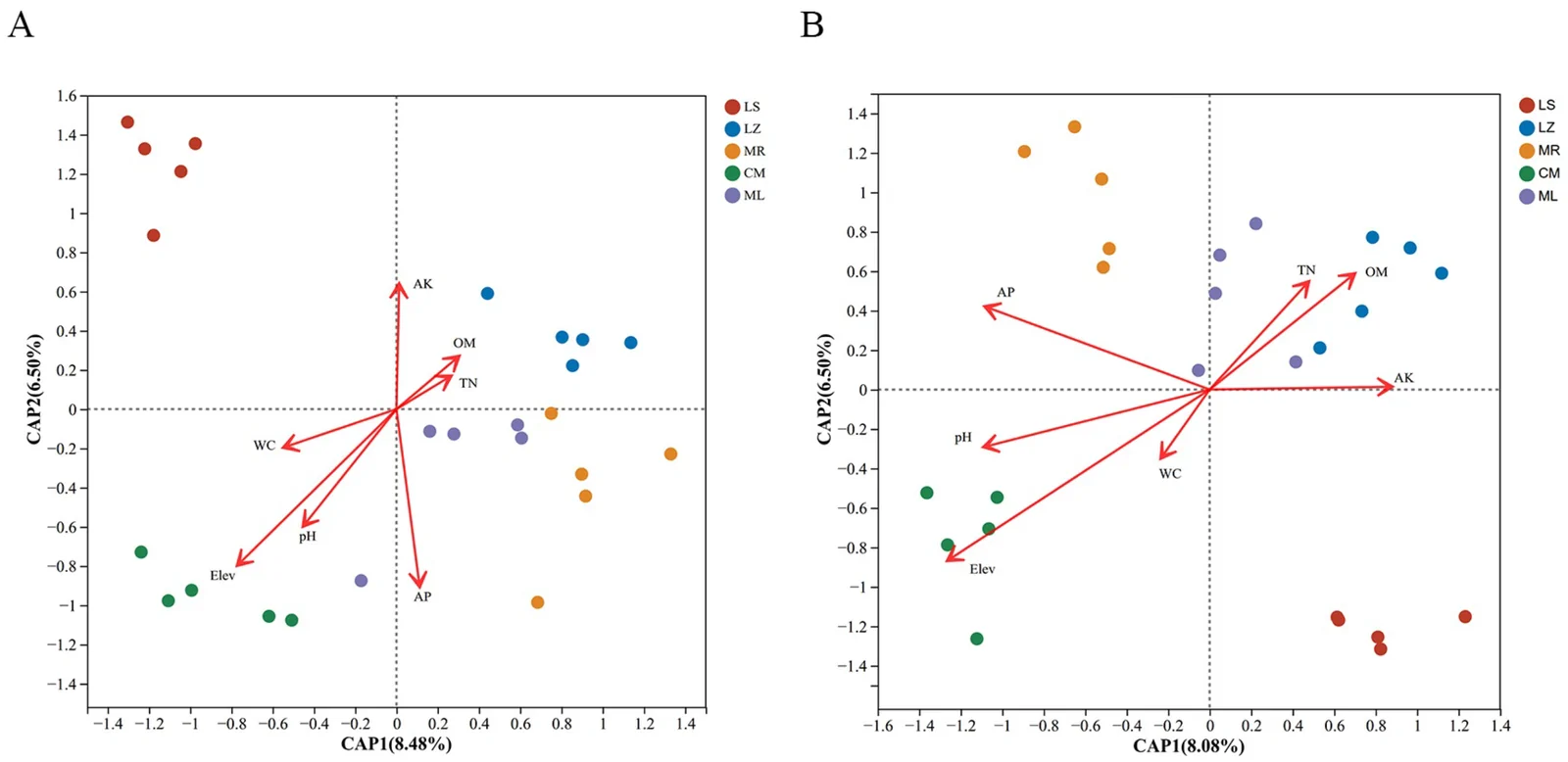
Distance-based redundancy analysis of rhizosphere bacterial (**A**) and fungal (**B**) communities constrained by soil properties and elevation AP, available phosphorus; AK, available potassium; SOM, soil organic matter; TN, total nitrogen; WC, water content; Elev, elevation.

db-RDA indicated associations between soil properties, elevation, and rhizosphere community structure (Figure 7). For bacteria, CAP1 and CAP2 explained 8.48% and 6.50% of the variation, respectively. LS, LZ, and CM samples were separated in ordination space; LZ was oriented toward SOM and TN, whereas CM was oriented toward pH and elevation, suggesting associations between bacterial differentiation and both nutrient conditions and geographic background. For fungi, CAP1 and CAP2 explained 8.08% and 6.50% of the variation, respectively, and site groups were clearly separated. LZ was oriented toward SOM and TN, CM toward pH, water content, and elevation, and MR toward AP. These patterns suggest that fungal community differentiation was associated with soil nutrients, pH, water availability, and elevation. However, the relatively low variance explained by the constrained axes indicates that measured environmental variables accounted for only part of the observed community variation and that unmeasured environmental factors and rhizosphere selection may also contribute.

### 3.8. Associations Between Core Genera, α Diversity, and Soil Properties

**Figure 8:**
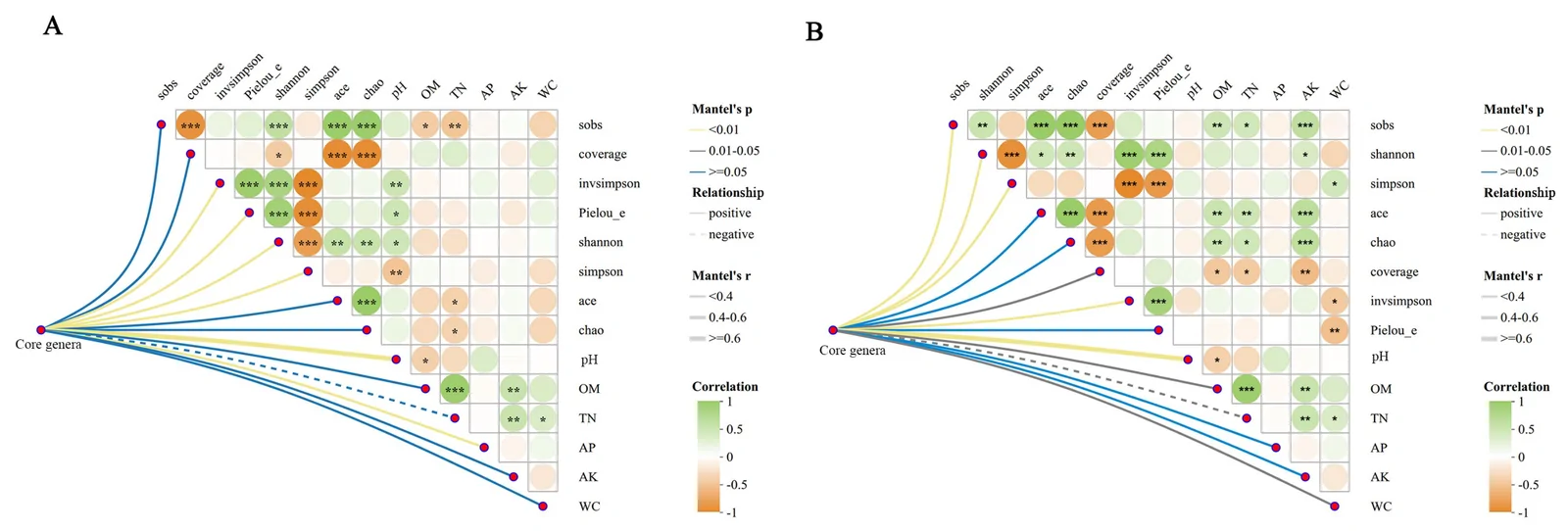
Associations among core bacterial (**A**) and fungal (**B**) genera, α-diversity indices, and soil physicochemical properties. * p<0.05, ** p<0.01, and *** p<0.001.

Spearman correlation heatmaps showed correlations among α-diversity indices and soil properties, while Mantel tests evaluated associations between environmental variables and core-genus community matrices (Figure 8 and Table 4). Soil pH was strongly associated with both core bacterial and core fungal communities. AP was strongly associated with core bacterial communities and significantly associated with core fungal communities, whereas AK was significantly associated only with core fungal communities. SOM, TN, and water content were not significantly associated with either core community. Richness estimators (observed OTUs, ACE, and Chao1) were positively intercorrelated, and SOM, TN, and AK also showed significant covariation. These results identify pH and AP as the soil variables most consistently associated with core-genus differentiation.

Mantel tests showed that pH was strongly associated with both core bacterial and fungal communities. AP was strongly associated with the core bacterial community and significantly associated with the core fungal community, whereas AK was significantly associated only with the core fungal community. SOM, TN, and water content were not significantly associated with either core community.

## 4. Discussion

This study first examined whether rhizosphere bacterial and fungal communities of *Paeonia ludlowii* exhibit concordant spatial responses following the transition from native to different introduced habitats. Both microbial groups showed significant differentiation among sites, with particularly pronounced separation between the Lhasa introduction site (LS) and the native sites. Previous studies have documented diverse rhizosphere fungal communities associated with *P. ludlowii* and suggested that habitat conditions may influence their community composition [26]. Studies of other plant species have also shown that changes in the rhizosphere environment can be accompanied by substantial shifts in microbial community structure and co-occurrence relationships [27]. In the present study, bacterial and fungal communities not only exhibited significant spatial differentiation independently but also showed strongly concordant patterns based on Mantel and Procrustes analyses. These statistical associations indicate that the two microbial groups exhibited broadly parallel patterns of community differentiation across the sampling sites. Such concordance may be related to their responses to partly shared environmental and host-associated conditions [11,12]; however, the present analyses do not identify the specific mechanisms responsible for these parallel patterns.

Despite their concordant spatial differentiation, bacterial and fungal communities differed in both the magnitude and nature of their responses, representing one of the central findings of this study. Bacterial Observed OTUs and Chao1 richness did not differ significantly among sites, whereas community composition varied markedly, indicating substantial compositional turnover despite relatively stable richness. In contrast, fungal communities showed more pronounced changes. Both Observed OTUs and Chao1 richness were significantly lower at LS, and the proportion of fungal OTUs shared between native and introduced habitats was substantially lower than that observed for bacteria. Community divergence analysis further reinforced this contrast: when native communities were used as the reference, fungal communities at the introduced sites showed greater overall divergence than bacterial communities. Previous studies have reported that bacterial and fungal communities can respond differently to environmental variation [28], potentially because of differences in their ecological characteristics and interactions with plants and soils. However, these mechanisms were not directly evaluated in the present study. Accordingly, our results demonstrate contrasting response patterns between bacterial and fungal communities, but do not establish the ecological mechanisms underlying these differences.

Environmental association analyses further indicated that the spatial differentiation of rhizosphere bacterial and fungal communities was associated with variation in soil conditions. Soil pH showed strong associations with both microbial groups, while available phosphorus (AP) was also significantly associated with variation in bacterial and fungal community composition. Previous research has shown that environmental gradients can be associated with shifts in the composition and functional differentiation of plant rhizosphere microbiomes [29]. Changes in environmental conditions can also be accompanied by changes in rhizosphere microbial community composition and microbial interactions [30]. In the present study, several soil properties, including pH, SOM, TN, and AP, differed among sites. LS was characterized by relatively high soil pH and simultaneously exhibited reduced fungal richness and greater fungal divergence from native assemblages. These coincident patterns suggest that local soil conditions may contribute to the observed community differences, but they do not establish a causal relationship.

Moreover, the first two constrained axes of the db-RDA explained only a limited proportion of the total community variation, indicating that the measured environmental variables accounted for only part of the observed differentiation. Other factors, including climatic conditions, plant growth status, root exudates, regional microbial pools, and long-term cultivation management, may also contribute to rhizosphere microbial reorganization. Accordingly, pH and AP should be interpreted as environmental variables that were consistently associated with community differentiation in this dataset, rather than as key determinants or direct drivers of microbial community change.

Taken together, the present study shows that rhizosphere bacterial and fungal communities of *P. ludlowii* exhibit broadly concordant spatial differentiation but differ in the extent of their departure from native community states. Fungal communities showed greater overall divergence from native assemblages, highlighting the importance of considering bacterial and fungal components jointly when evaluating rhizosphere microbiome changes associated with the introduction and ex situ conservation of endangered plants. Several limitations should be considered when interpreting these findings. First, this study was based on a single-period spatial sampling design, and the native and introduced populations occurred at geographically distinct sites that also differed in soil properties, elevation, local environmental conditions, and management history. Consequently, the effects associated with habitat transition cannot be fully disentangled from geographic and site-specific influences, and the observed community differences should therefore be interpreted as associations rather than as direct causal effects of introduction. Second, the present study focused primarily on soil physicochemical properties and did not incorporate host-related factors such as plant growth, physiological status, root traits, or root exudates. In addition, amplicon sequencing primarily characterizes taxonomic patterns and does not directly establish whether the observed compositional differences correspond to changes in microbial functions. Future studies incorporating replicated native and introduced populations, temporal monitoring, host functional traits, and metagenomic or metatranscriptomic approaches will be needed to disentangle habitat-transition effects from geographic variation and to clarify the ecological and functional consequences of rhizosphere microbiome reorganization.

## 5. Conclusions

The central finding of this study is that rhizosphere bacterial and fungal communities of *Paeonia ludlowii* exhibit broadly concordant spatial differentiation but differ in the extent of their departure from native community states, with fungal communities showing greater overall divergence. This contrast indicates that responses of the rhizosphere microbiome to introduction cannot be adequately represented by a single microbial group. From a conservation perspective, jointly considering bacterial and fungal communities may provide a more comprehensive assessment of belowground ecological changes accompanying the introduction and ex situ conservation of endangered plants. Because habitat transition could not be separated completely from geographic and site-specific variation in the present spatial design, these patterns should be interpreted as associations rather than causal effects of introduction. Future studies incorporating replicated introduction systems, temporal monitoring, host functional traits, and microbial functional analyses will be important for determining whether the observed community changes influence plant establishment and adaptation in introduced habitats.

## Figures and Tables

**Table 1:** Sampling sites and environmental characteristics.

Code	Category	Location	Elevation (m)	Main Environmental Characteristics
MR	Native	Qunigongga Village, Mirui Township, Nyingchi	3044	Slope in the Yarlung Zangbo River valley with mixed native shrub and herbaceous vegetation
CM	Native	Caimen Village, Zaxiraodeng Township, Milin	2900	Open valley on the north bank of the Yarlung Zangbo River; alluvial sandy loam; slight anthropogenic disturbance
ML	Native	Qinmaduo, Milin Town, Milin	3120	Mountain-slope forest margin on the north bank of the Yarlung Zangbo River; colluvial gravelly soil; shallow soil layer; moderate-to-high grazing disturbance
LZ	Introduced	Botanical Garden, Plateau Ecology Institute, Tibet Agricultural and Animal Husbandry University	2970	Local introduction site on a terrace of the Nyang River; alluvial sandy loam; deep soil layer
LS	Introduced	Agricultural Science Research Station, Tibet Academy of Agricultural and Animal Husbandry Sciences	3568	ex situ introduction site in an agricultural experimental field with a long history of soil management

**Table 2:** Physicochemical properties of rhizosphere soil across sampling sites.

Site	pH	SOM (g kg^−1^)	TN (g kg^−1^)	AP (mg kg^−1^)	AK (mg kg^−1^)	Gravimetric Water Content (%)
MR	6.98±0.38 b	40.19±23.50 b	1.93±1.01 b	8.71±3.67 c	412.31±177.94 a	6.32±3.40 a
CM	7.40±0.35 ab	77.61±25.70 a	4.02±1.37 a	24.83±9.99 c	374.29±205.48 a	8.65±2.25 a
ML	6.95±0.19 b	52.38±22.82 ab	2.56±1.11 ab	174.98±59.73 a	362.53±150.33 a	6.99±3.28 a
LZ	6.27±0.30 c	56.55±14.55 ab	2.49±0.73 ab	24.53±9.24 c	231.77±102.00 a	8.59±3.25 a
LS	7.94±0.15 a	27.65±1.04 b	1.60±0.14 b	116.22±32.36 b	165.51±29.26 a	9.56±2.53 a

Note: Values are presented as mean ± SD (n=5). Different lowercase letters within the same column indicate significant differences at p<0.05.

**Table 3:** α-diversity indices of rhizosphere bacterial and fungal communities across sampling sites.

Domain	Site	Observed OTUs	Chao1	Good’s Coverage	Shannon	Inverse Simpson	Pielou’s Evenness
Bacteria	MR	3780.60±116.57 a	4687.07±151.76 a	0.97716±0.00108 a	6.56±0.08 a	155.72±31.20 b	0.7969±0.0084 a
	CM	3345.80±234.50 a	4138.48±254.49 a	0.98057±0.00134 a	6.59±0.12 a	228.91±60.03 a	0.8119±0.0086 a
	ML	3358.60±141.20 a	4219.86±221.95 a	0.97970±0.00166 a	6.37±0.15 a	140.63±76.15 b	0.7841±0.0218 b
	LS	3698.40±308.95 a	4481.09±436.63 a	0.97907±0.00284 a	6.77±0.17 a	288.33±89.32 a	0.8242±0.0119 a
	LZ	3454.40±498.80 a	4298.93±567.95 a	0.97941±0.00284 a	6.44±0.37 a	153.39±71.13 b	0.7912±0.0335 a
Fungi	MR	571.40±33.62 a	604.45±37.96 a	0.99878±0.00027 a	4.17±0.16 a	25.71±6.05 a	0.6573±0.0274 a
	CM	593.40±95.10 a	626.63±103.44 a	0.99884±0.00034 a	4.16±0.26 a	24.78±4.51 a	0.6529±0.0301 a
	ML	582.60±126.06 a	620.63±140.19 a	0.99874±0.00086 a	4.19±0.62 a	28.44±16.85 a	0.6606±0.0980 a
	LS	311.40±93.66 b	324.64±98.73 b	0.99954±0.00034 a	3.60±0.41 a	14.06±5.81 a	0.6324±0.0710 a
	LZ	416.60±119.29 a	434.74±124.34 a	0.99926±0.00038 a	4.03±0.30 a	25.71±17.23 a	0.6771±0.0924 a

Note: Values are means ± standard deviations (n=5). Different lowercase letters within a column indicate significant differences at p<0.05.

**Table 4:** Mantel correlations of core bacterial and fungal communities with soil properties.

Variable	Core Bacterial Genera		Core Fungal Genera	
	*r*	*p*	*r*	*p*
pH	0.752	0.001 **	0.629	0.001 **
SOM	0.084	0.118	0.076	0.158
TN	−0.053	0.727	−0.067	0.767
AP	0.240	0.002 **	0.158	0.011 *
AK	0.075	0.142	0.163	0.040 *
Water content	0.050	0.172	0.071	0.141

Note: *r* denotes the Mantel correlation coefficient. *p* values were obtained from 999 permutations. * *p* <0.05; ** *p*<0.01.

## Data Availability

The raw sequencing data generated in this study have been deposited in the NCBI Sequence Read Archive (SRA) database under BioProject accession number PRJNA1498593. The OTU tables, environmental data, and other datasets supporting the findings of this study are available from the corresponding author upon reasonable request.

## Abbreviations

The following abbreviations are used in this manuscript:

OTU	Operational taxonomic unit
SOM	Soil organic matter
TN	Total nitrogen
AP	Available phosphorus
AK	Available potassium
PCoA	Principal coordinate analysis
PERMANOVA	Permutational multivariate analysis of variance
LEfSe	Linear discriminant analysis effect size
db-RDA	Distance-based redundancy analysis
ITS	Internal transcribed spacer
SRA	Sequence Read Archive

## References

[1] Díaz S., Settele J., Brondízio E.S., Ngo H.T., Agard J., Arneth A., Balvanera P., Brauman K.A., Butchart S.H.M., Chan K.M.A. (2019). Pervasive human-driven decline of life on Earth points to the need for transformative change. Science.

[2] Brondízio E.S., Settele J., Díaz S., Ngo H.T., IPBES (2019). Global Assessment Report on Biodiversity and Ecosystem Services of the Intergovernmental Science-Policy Platform on Biodiversity and Ecosystem Services.

[3] Berg G., Dorador C., Egamberdieva D., Kostka J.E., Ryu C.-M., Wassermann B. (2024). Shared governance in the plant holobiont and implications for one health. FEMS Microbiol. Ecol..

[4] Vandenkoornhuyse P., Quaiser A., Duhamel M., Le Van A., Dufresne A. (2015). The importance of the microbiome of the plant holobiont. New Phytol..

[5] Qu Q., Zhang Z., Peijnenburg W.J.G.M., Liu W., Lu T., Hu B., Chen J., Chen J., Lin Z., Qian H. (2020). Rhizosphere microbiome assembly and its impact on plant growth. J. Agric. Food Chem..

[6] Peddle S.D., Hodgson R.J., Borrett R.J., Brachmann S., Davies T.C., Erickson T.E., Liddicoat C., Muñoz-Rojas M., Robinson J.M., Watson C.D. (2025). Practical applications of soil microbiota to improve ecosystem restoration: Current knowledge and future directions. Biol. Rev..

[7] Trivedi P., Leach J.E., Tringe S.G., Sa T., Singh B.K. (2020). Plant–microbiome interactions: From community assembly to plant health. Nat. Rev. Microbiol..

[8] Fitzpatrick C.R., Salas-González I., Conway J.M., Finkel O.M., Gilbert S., Russ D., Teixeira P.J.P.L., Dangl J.L. (2020). The plant microbiome: From ecology to reductionism and beyond. Annu. Rev. Microbiol..

[9] Peixoto R.S., Voolstra C.R., Sweet M., Duarte C.M., Carvalho S., Villela H., Lunshof J.E., Gram L., Woodhams D.C., Walter J. (2022). Harnessing the microbiome to prevent global biodiversity loss. Nat. Microbiol..

[10] Choi S., Lee H.-S. (2024). Interplay between plants and microbial communities: Insights from holobionts and environmental interactions. Phyton-Int. J. Exp. Bot..

[11] Zeng Q., Hu H.-W., Ge A.-H., Xiong C., Zhai C.-C., Duan G.-L., Han L.-L., Huang S.-Y., Zhang L.-M. (2025). Plant–microbiome interactions and their impacts on plant adaptation to climate change. J. Integr. Plant Biol..

[12] Li Y., Qu N., Li S., Zhou H., Yue M. (2025). Ecological mechanisms of microbial assembly in clonal plant *Glechoma longituba*: Soil Endosphere. Appl. Environ. Microbiol..

[13] Xun W., Shao J., Shen Q., Zhang R. (2021). Rhizosphere microbiome: Functional compensatory assembly for plant fitness. Comput. Struct. Biotechnol. J..

[14] Park I., Seo Y.-S., Mannaa M. (2023). Recruitment of the rhizo-microbiome army: Assembly determinants and engineering of the rhizosphere microbiome as a key to unlocking plant potential. Front. Microbiol..

[15] Causevic S., Tackmann J., Sentchilo V., Malfertheiner L., von Mering C., van der Meer J.R. (2025). Habitat filtering more than microbiota origin controls microbiome transplant outcomes in soil. ISME J..

[16] Chen W., Zhu B., Yang C., Wei C., He Y., Zheng L., Liu X., Yang J., Tedersoo L., Lu X. (2025). Decoupling responses of phyllosphere and rhizosphere bacterial communities to spatiotemporal environmental changes. Glob. Change Biol..

[17] Schaefer S.R., Montaño-López F., Holland-Moritz H., Hicks Pries C., Ernakovich J.G. (2025). Rhizosphere bacteria and fungi are differentially structured by host plants, soil mineralogy, and ectomycorrhizal communities in the Alaskan Tundra. Arct. Sci..

[18] Liu Q., Dai H., Cheng H., Shao G., Wang L., Zhang H., Gao Y., Liu K., Xie X., Gong J. (2025). Rhizosphere-associated bacterial and fungal communities of two maize hybrids under increased nitrogen fertilization. Front. Plant Sci..

[19] Lu Y., Zhang E., Hong M., Yin X., Cai H., Yuan L., Yuan F., Li L., Zhao K., Lan X. (2020). Analysis of endophytic and rhizosphere bacterial diversity and function in the endangered plant *Paeonia ludlowii*. Arch. Microbiol..

[20] Gao D., Wu L., Meng F., Yuan T. (2022). Rhizosphere microbial diversity and community structure of *Paeonia ludlowii* Introd. Area. J. Northeast Agric. Univ..

[21] Qiao H., Gao D., Yuan T. (2023). Differences in rhizosphere soil fungal communities of wild and cultivated *Paeonia ludlowii* Species. Front. Plant Sci..

[22] Chen S., Zhou Y., Chen Y., Gu J. (2018). fastp: An ultra-fast all-in-one FASTQ preprocessor. Bioinformatics.

[23] Magoč T., Salzberg S.L. (2011). FLASH: Fast length adjustment of short reads to improve genome assemblies. Bioinformatics.

[24] Edgar R.C. (2013). UPARSE: Highly accurate OTU sequences from microbial amplicon reads. Nat. Methods.

[25] Wang Q., Garrity G.M., Tiedje J.M., Cole J.R. (2007). Naive Bayesian classifier for rapid assignment of rRNA sequences into the new bacterial taxonomy. Appl. Environ. Microbiol..

[26] Lu Y.Z., Zhang E.H., Yin X., Cai H., Yuan L., Li L.Q., Zhao K.T., Lan X.Z. (2020). Community diversity of endophytic fungi and rhizosphere soil fungi associated with the endangered Tibetan plant *Paeonia ludlowii*. J. Biol..

[27] He Y.H., Huang Y.H., Lu R.M., Yang R.S., Wei Y.N., Liang W.H. (2024). Responses of avocado rhizosphere soil microbial community and its co-occurrence network to root rot disease. J. Cent. South Univ. For. Technol..

[28] Loiko N., Islam M.N. (2024). Plant–Soil Microbial Interaction: Differential Adaptations of Beneficial vs. Pathogenic Bacterial and Fungal Communities to Climate-Induced Drought. Agronomy.

[29] Li J., Zhang H., Wang T., Peng L., Huang K., Tang X., Li W., Xu Z., Li C., Chen F. (2025). Functional differentiation of industrial hemp rhizosphere microbiome along environmental gradients. Front. Plant Sci..

[30] Li R., Jiao H., Sun B., Song M., Yan G., Bai Z., Wang J., Zhuang X., Hu Q. (2024). Understanding Salinity-Driven Modulation of Microbial Interactions: Rhizosphere versus Edaphic Microbiome Dynamics. Microorganisms.

